# Editorial: Debates in regenerative dentistry

**DOI:** 10.3389/fdmed.2024.1412613

**Published:** 2024-05-01

**Authors:** Martha J. Somerman, Paul Sharpe, Pamela C. Yelick, Sema S. Hakki

**Affiliations:** ^1^ Field Chief Editor, Frontiers in Dental Medicine, Chevy Chase, MD, United States; ^2^ CCRB, King’s College London, London, United Kingdom; ^3^ School of Dental Medicine, Tufts University, Boston, MA, United States; ^4^ Faculty of Dentistry, Selcuk University, Konya, Türkiye

**Keywords:** regeneration, debates, growth factors, periodontal disease, scaffolds, wound healing

**Editorial on the Research Topic** Debates in regenerative dentistry

The classical concept of tissue regeneration/restoration involves a delivery vehicle (biomaterial scaffold) combined with inductive factors (growth factors, proteins, extracellular matrix components and /or small molecules) and in some applications, cells. Fundamental issues with these approaches for dental treatments are the prohibitive costs and concerns of using in vitro expanded cells and highly purified growth factors for applications in humans.

Frontiers, to facilitate debate and discussion amongst the community, launched a series of Research Topics focusing on vital questions related to the sections interest, utilizing a debates/discussion type platform. In this new Topic, “Debates in Regenerative Dentistry”, a small clinical trial study, two perspectives—one addressing the application of platelet-derived proteins in periodontal tissue regeneration and the other, a gathering of academicians and clinicians discussing methods and procedures for oral tissue regeneration, from research to clinical practice -, and an original research article describing the development of a new delivery nanomaterial with specific features for craniofacial tissue regeneration, were peer reviewed and accepted for publication.

The article by Pitzurra et al., reported on a small clinical trial, that compared the use of platelet-rich fibrin to treat periodontal disease with existing treatments involving enamel matrix derivative (EMD) and open flap debridement (OFD). The results indicated platelet-rich fibrin performed slightly better than EMD, and that OFD treated regression but delayed wound healing. Clearly, further studies are required that include larger patient numbers and combined approaches. In a perspective written by Maria Geisinger, the potential of “growth factors” and other signaling molecules to stimulate periodontal tissue regeneration were presented, including a summary of studies utilizing human recombinant platelet-derived growth factors.

In the perspective by Puterman et al., a video format was used by InDent Research, an established five-member group consisting of researchers, academicians and clinicians, to discuss current therapies for treating periodontal diseases and future research needed to advance predictable regenerative therapies. All agreed that while improvements have been made in the materials available for clinical use, more research is needed to improve scaffold design, materials, and tools and technologies available to dental clinicians.

Finally in an original research study, Woodbury et al. documented the synthesis and preliminary testing of a novel temperature responsive nanofiber material that in mice permits vascularization and bone regeneration.

The field of Regenerative Dentistry aims to deliver a new generation of dental therapies based on evidence-based research, and the last decade has seen major developments in understanding the biology of dental tissues and how pathways might be manipulated to promote resident cells to generate new tissues. Current efforts focus on developing cell-free based approaches for regenerative dental/periodontal therapies including: isolated nano sized extracellular exosomal vesicles; and engineered exosomes specifically designed to target specific cell types. In turn, appropriate carrier biomaterials for the exosomes will need to be developed to ensure the success of these approaches.

Although of great potential, major challenges remain with respect to effectively informing oral health care providers, the dental commercial sector, and the general public, that regenerative dental therapies can be significantly better than existing treatments, and can be delivered safely and in a cost-effective manner. We feel certain that evidence-based research, definitive clinical trials, and effective education of dental pre- and postdoctoral students and oral health care providers will result in the availability of reliable Regenerative Dentistry clinical therapies in the near future.

Sema S. Hakki, Paul Sharpe, Martha J. Somerman, Pamala C. Yelick

